# Role of Estimated Glucose Disposal Rate in Staging and Death Risk of Cardiovascular-Kidney-Metabolic Syndrome: Insights from NHANES 1999-2018

**DOI:** 10.7150/ijms.116708

**Published:** 2025-08-16

**Authors:** Jin Ke, Shuang Wu, Hongyang Xu, Fengming Liang, Jing Tian, Qiuhui Wang, Yang Chen

**Affiliations:** 1 Department of Critical Care Medicine, The Affiliated Wuxi People's Hospital of Nanjing Medical University, Wuxi Medical Center, Nanjing Medical University, No. 299 Qingyang Road, Wuxi 214023, China.; 2 National Center for Cardiovascular Disease, Fuwai Hospital, Chinese Academy of Medical Sciences and Peking Union Medical College, Beijing, People's Republic of China.; 3 National Clinical Research Center of Cardiovascular Diseases, National Center for Cardiovascular Disease, Fuwai Hospital, Chinese Academy of Medical Sciences and Peking Union Medical College, Beijing, People's Republic of China.; 4 Liverpool Centre for Cardiovascular Science at University of Liverpool, Liverpool John Moores University and Liverpool Heart and Chest Hospital, Liverpool, United Kingdom.; 5 Department of Cardiovascular and Metabolic Medicine, Institute of Life Course and Medical Sciences, University of Liverpool, Liverpool, United Kingdom.

**Keywords:** Estimated glucose disposal rate, Cardiovascular-kidney-metabolic syndrome, Insulin resistance, Death, NHANES

## Abstract

**Background:** The concept of cardiovascular-kidney-metabolic syndrome (CKM) was recently proposed by the American Heart Association. Insulin resistance (IR) is closely linked to metabolic disorders, chronic kidney disease, and cardiovascular disease, which are the key components of CKM. As a surrogate IR marker, estimated glucose disposal rate (eGDR) may help identify high-risk patients. However, the specific role of eGDR in CKM progression and outcomes remains undefined. We aimed to evaluate the associations between eGDR and CKM progression, as well as its association with death in patients with CKM.

**Methods:** Data was obtained from the National Health and Nutrition Examination Survey 1999-2018. Adults aged ≥ 20 years with complete data on CKM components and eGDR were included. Study outcomes were CKM progression and death outcomes. Multinomial logistic regression was used to evaluate the association between eGDR and CKM staging. Kaplan-Meier curves and Cox proportional hazard models assessed death outcomes, with restricted cubic splines exploring non-linear relationships. Stratified and sensitivity analyses tested the robustness of results. The predictive performance of eGDR was compared with the Homeostasis Model Assessment of Insulin Resistance and triglyceride-glucose index for death outcomes.

**Results:** 29,290 participants were included (median age: 53.00 years, 51.96% males), with 27,769 classified as having CKM. Higher eGDR was also associated with lower odds of progression to advanced CKM stages. In CKM patients, over a median follow-up of 8.92 years, 4,926 deaths occurred (17.7%), with 1,330 (4.8%) cardiovascular deaths and 3,596 (12.9%) non-cardiovascular deaths. Compared with the lowest eGDR quartile, CKM patients in the highest quartile had lower risk of all-cause death (HR=0.59, 95%CI: 0.52-0.66), cardiovascular death (HR=0.52, 95%CI: 0.41-0.66), and non-cardiovascular death (HR=0.60, 95%CI: 0.53-0.69) (all *P*<0.001). Non-linear relationships between eGDR and death outcomes were observed (all *P for nonlinear*<0.001). Subgroup and sensitivity analyses confirmed the robustness of these associations. Additionally, eGDR predicted death in CKM patients better than other IR markers.

**Conclusions:** Our findings support the utility of eGDR as a risk stratification tool in CKM populations. Lower eGDR levels were associated with more advanced CKM stages and higher long-term mortality, suggesting its potential role in identifying high-risk individuals.

## Introduction

CKM is a systemic disease characterized by the pathological interactions between metabolic risk factors, chronic kidney disease (CKD), and the cardiovascular system, leading to multi-organ failure and higher incidences of cardiovascular events and all-cause death 1. According to the American Heart Association (AHA), the global prevalence of CKM is high, particularly among populations with adverse social determinants of health, such as low income and low educational levels 1, 2. CKM patients typically suffer from two or three diseases, including type 2 diabetes mellitus (T2DM), CKD, and cardiovascular disease (CVD), with a 2-3-fold higher all-cause death risk compared to those with a single disease 3. In CVD patients with coexisting T2DM and CKD, the risk of cardiovascular death is seven times higher 4. Due to the rising global incidence of CKM and a reduction in life expectancy of 30%-45% compared to healthy populations 5, it has become a significant public health challenge.

Insulin resistance (IR), a key contributor to cardiometabolic dysfunction, underlies the pathogenesis of obesity, diabetes, and hypertension, which are core components of CKM 6, 7. While markers such as Homeostasis Model Assessment of Insulin Resistance (HOMA-IR) and the triglyceride-glucose index (TyG) are commonly used to assess IR 8, 9, the estimated glucose disposal rate (eGDR) integrates multiple metabolic parameters and may offer a more comprehensive evaluation 10. Given the central role of IR in CKM-related complications, particularly through mechanisms such as ectopic fat accumulation, oxidative stress, and fibrosis 11-13, a better understanding of its clinical relevance is needed. However, the association between eGDR and CKM staging, as well as its prognostic significance for mortality, remains unclear.

This study primarily examines two-fold key aspects: (1) analyzing the association between eGDR and CKM staging; (2) evaluating the link between eGDR and death risks in CKM patients.

## Methods

### Data sources

This study utilized data from the National Health and Nutrition Examination Survey (NHANES) 1999-2018. Conducted by the Centers for Disease Control and Prevention, NHANES assesses the health and nutritional status of the U.S. population, which received approval from the National Center for Health Statistics Ethics Review Board. As all data were anonymised, this study was exempt from ethical review and informed consent requirements. It adheres to the Strengthening the Reporting of Observational Studies in Epidemiology (STROBE) guidelines (Supplementary Table S1). This study includes ten consecutive survey cycles from 1999 to 2018, the preliminary screening was conducted on 55,081 adult subjects (aged ≥ 20 years), outlining the study population selection process. Exclusion criteria: (1) 17,949 participants without complete CKM-related data; (2) 2,162 without complete eGDR-related data; (3) 626 participants who were pregnant; (4) 5,035 with missing baseline information; (5) 19 without complete death follow-up (Supplementary Fig. S1).

### Definitions of the eGDR

The eGDR was calculated using the formula: 21.158 - (0.09 × waist circumference [WC] [cm]) - (3.407 × hypertension status [yes = 1, no = 0]) - (0.551 × hemoglobin A1c [%]) 14. Subjects were divided into four groups based on the quartile values of eGDR: quartile 1 (Q1), eGDR < 4.72; quartile 2 (Q2), 4.72 ≤ eGDR < 6.00; quartile 3 (Q3), 6.00 ≤ eGDR < 7.83; and quartile 4 (Q4), eGDR ≥ 7.83.

### Study Outcomes

The primary outcomes were CKM staging and death outcomes (including all-cause death, cardiovascular death, and non-cardiovascular death). CKM was defined according to the criteria outlined in Supplementary Table S2. Additionally, the AHA PREVENT equations were applied to calculate the 10-year risk of CVD, as shown in Supplementary Table S3 15. Based on Kidney Disease: Improving Global Outcomes criteria, kidney function was classified 16. According to the AHA criteria, CKM was divided into four distinct stages, as shown in Supplementary Table S4 17. The data processing methodology in this study follows the same approach as our previously published NHANES-based CKM analysis 18. Death outcome data were retrieved from the Centers for Disease Control and Prevention database, reflecting follow-up through the end of 2019. The underlying causes of death were coded using the 10th edition of the International Statistical Classification of Diseases and Related Health Problems. Follow-up duration was calculated from the date of the baseline interview to the occurrence of death or the last recorded follow-up.

### Covariates

The covariates in this analysis included demographic information (such as age, poverty income ratio [PIR]), physical characteristics (such as waist circumference [WC], height), health-related behaviors (such as smoking status, physical activity), biological indicators (such as hemoglobin A1c, total cholesterol). Body mass index (BMI) was calculated as weight (kg) divided by height squared (m^2^). Specimens of blood and urine were obtained using standardised protocols, then handled, preserved, and analysed at the University of Minnesota (Minneapolis, MN). Participants self-reported their race and ethnicity, categorised into non-Hispanic Black, non-Hispanic White, Hispanic, Mexican American, and others. Participants' marital status was grouped as unmarried, married, or divorced. Based on self-reported cigarette use, participants were defined as never smokers (<100 cigarettes in total), former smokers (≥100 cigarettes but not currently smoking), or current smokers (≥100 cigarettes and actively smoking). The Chronic Kidney Disease Epidemiology Collaboration (CKD-EPI) equation was employed to estimate glomerular filtration rate 19. The ratio of urine albumin to creatinine was computed as the ratio of urine albumin (µg/mL) to urine creatinine (mg/dL), then multiplied by 100 18.

### Statistical analysis

No missing values in this study. Statistical analyses followed CDC guidelines. For continuous variables, the interquartile range (IQR) was reported. Group differences were analysed using Kruskal-Wallis tests. Categorical variables are presented as counts and percentages and were assessed using Fisher's exact test or Chi-squared test.

Given the partial overlap between variables used to calculate eGDR and those defining CKM stages, potential collinearity was assessed. Spearman's rank correlation coefficient between the two variables was -0.382, and the variance inflation factor from a linear regression model including both terms was 1.142, indicating no concerning multicollinearity.

Although the staging of CKM was treated as an ordinal variable, the parallel line assumption was violated, leading to the use of multinomial logistic regression to assess the link between eGDR and staging of CKM, fully adjusted for age, sex, BMI, race/ethnicity, marital status, education level, physical activity, smoking status, PIR, and alcohol consumption, presenting as odds ratio (OR) and 95% confidence interval (CI). The cumulative incidence of death across different eGDR quartiles in CKM patients was shown using Kaplan-Meier survival curves, with group differences assessed by the Log-rank test. Three Cox proportional hazard models were used to examine the association between eGDR quartiles and death outcomes in CKM patients, expressing the results as hazard ratio (HR) and 95% CI. Model I was unadjusted; Model II was adjusted for age, sex, BMI, race and ethnicity; Model III was further adjusted based on Model II by including marital status, education level, physical activity, smoking status, PIR, and alcohol consumption. Restricted cubic spline (RCS) models were employed to assess potential nonlinear associations between the eGDR and death outcomes. Stratified regression modeling and interaction effect testing were conducted to investigate how demographic factors (age [<60 vs. ≥60 years], sex, race [White vs. non-White]), clinical parameters (BMI [≥30 vs. < 30 kg/m²]), and CKM staging (stages 1/2 vs. 3/4) modulated the relationship between eGDR and death risk.

Furthermore, three sensitivity analyses were conducted to validate the robustness of findings: (i) To account for the substantial confounding effect of malignancy on death outcomes, we reassessed the primary associations by removing CKM patients with documented history of cancer; (ii) To mitigate potential reverse causation bias, participants who experienced fatal events within the initial 24-month follow-up period were systematically removed, followed by reanalysis of eGDR-death relationships; (iii) Addressing the influence of systemic inflammation, we excluded cases with incomplete C-reactive protein (CRP) measurements and incorporated CRP as a covariate in multivariable Model III to verify the stability of eGDR's predictive capacity for death outcomes.

Finally, given the established roles of eGDR, TyG, and HOMA-IR as surrogate markers of insulin resistance, we further assessed their predictive value for death outcomes. Model discrimination capacity was determined by calculating the area under (AUC) the Receiver operating characteristic (ROC). Statistical comparisons of AUC values between models were performed using the DeLong test.

Statistical analyses were conducted using R software (version 4.4.2) and SPSS Statistics (version 27). All analyses employed two-tailed tests, with statistical significance set at *P* < 0.05.

## Results

### Baseline characteristics

The final analytical cohort included 29,290 participants (median age 53.0 years [IQR 39.0-66.0]; 51.96% male), comprising 27,769 individuals with CKM and 1,521 without. The median follow-up time was 9.08 years [IQR 4.92-13.58], with CKM patients followed for a median of 8.92 years [IQR 4.83-13.42] and non-CKM individuals for 10.92 years [IQR 6.83-15.75]. Baseline characteristics across CKM stages are summarized in Supplementary Table S5. CKM stage 2 accounted for the largest proportion of participants (all *P* < 0.001), who tended to have higher PIR, more advanced education, greater engagement in vigorous physical activity, and higher rates of smoking and alcohol consumption (all *P* < 0.001).

In Table 1, baseline characteristics are presented by eGDR quartiles within the CKM subgroup. Similarly, participants with higher eGDR values were more likely to have elevated PIR, higher education levels, greater physical activity, and a higher prevalence of smoking and alcohol use (all *P* <0.001). Additionally, there was a progressive reduction in the percentage of subjects at CKM stages 3/4 with increasing eGDR levels.

### Relationship between eGDR and CKM staging progression

**Figure 1:**
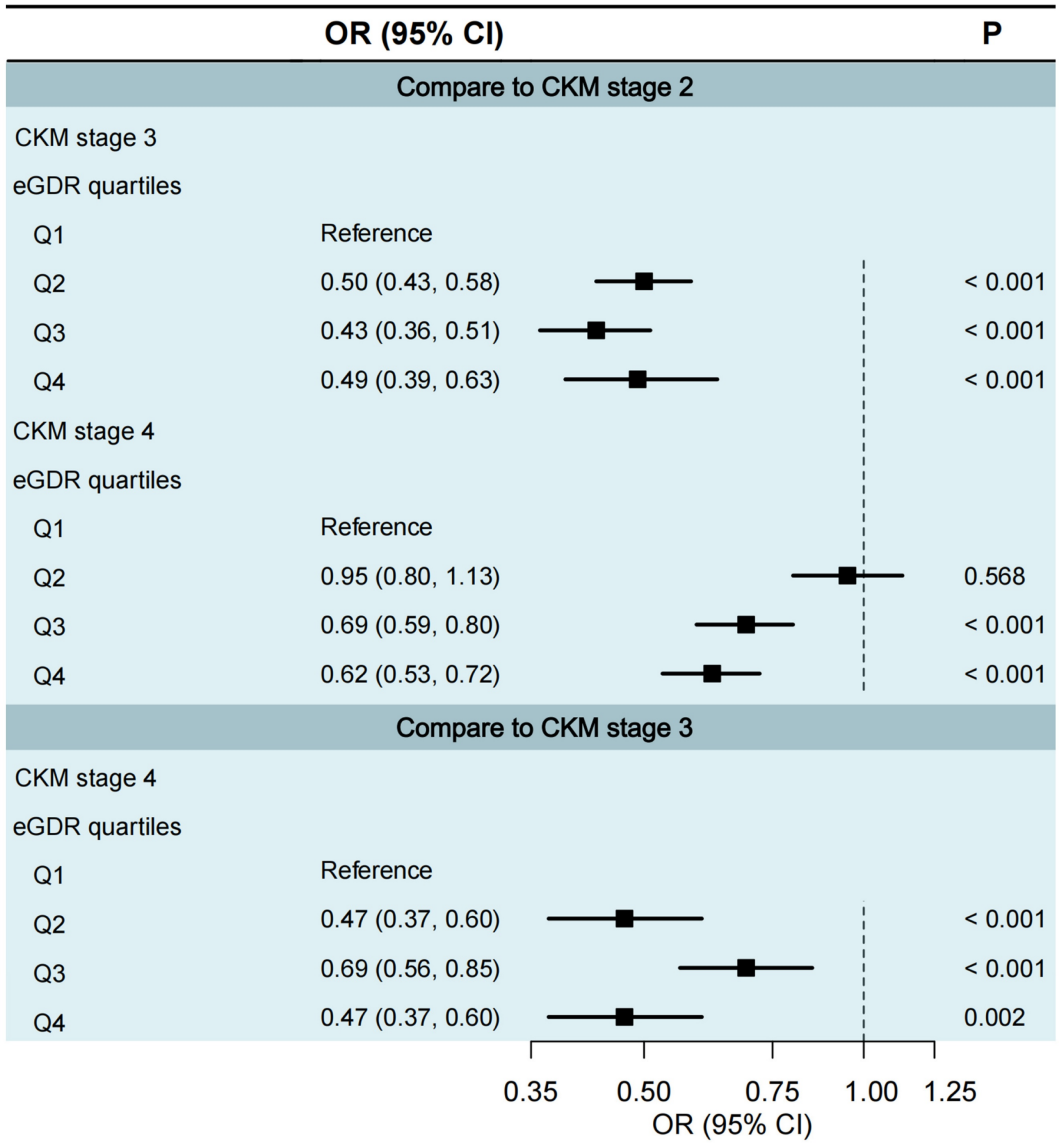
** Multinomial logistic regression of eGDR quartiles in relation to CKM staging.** Q1 <4.72, 4.72≤ Q2 <6.00, Q3: 6.00≤ Q3 <7.83, Q4 ≥7.83. CI, confidence interval; CKM, cardiovascular-kidney-metabolic syndrome; eGDR, estimated glucose disposal rate; OR, odds ratio.

We excluded CKM stages 0 and 1 from the multinomial logistic regression analysis was justified due to insufficient sample sizes in certain eGDR quartiles. Specifically, in CKM stage 0, the proportions of participants in eGDR Q1 to Q3 were all 0%; and in CKM stage 1, only 8 participants (0.3%) were in Q1 and 38 participants (1.2%) in Q2. Therefore, we only assessed cross-section staging through CKM stages 2 to 4 (Fig. 1). Compared to CKM stage 2, participants in higher eGDR quartiles had significantly lower odds of being in CKM stage 3 (Q1 as the reference group; Q2: OR = 0.50, 95% CI 0.43-0.58; Q3: OR = 0.43, 95% CI 0.36-0.51; Q4: OR = 0.49, 95% CI 0.39-0.63; all *P* < 0.001) and CKM stage 4 (Q1 as the reference group; Q3: OR = 0.69, 95% CI 0.59-0.80; Q4: OR = 0.62, 95% CI 0.53-0.72; both *P* < 0.001). Similarly, compared to CKM stage 3, higher eGDR quartiles were associated with significantly lower odds of being in CKM stage 4 (Q1 as reference; Q2: OR = 0.47, 95% CI 0.37-0.60, *P* < 0.001; Q3: OR = 0.69, 95% CI 0.56-0.85, *P* < 0.001; Q4: OR = 0.47, 95% CI 0.37-0.60, *P* = 0.002).

### Death outcomes distribution across eGDR quartiles and CKM stages

Supplementary Fig. S2 demonstrated inverse correlations between death outcomes and both CKM staging and eGDR quartiles over a median 8.92-year follow-up (all *P* < 0.001). The non-CKM group exhibited the lowest all-cause death (2.4%), contrasting with substantially elevated rates in advanced CKM stages (stage 3: 50.7%; stage 4: 41.6%). A comparable pattern was seen for cardiovascular and non-cardiovascular deaths. Additionally, as eGDR quartiles increased, all-cause death declined from 21.4% in Q1 to 9.9% in Q4. Cardiovascular death similarly decreased from 6.3% to 2.2%, and non-cardiovascular death from 15.1% to 7.7%.

### Relationship between eGDR and death outcomes in CKM patients

**Figure 2:**
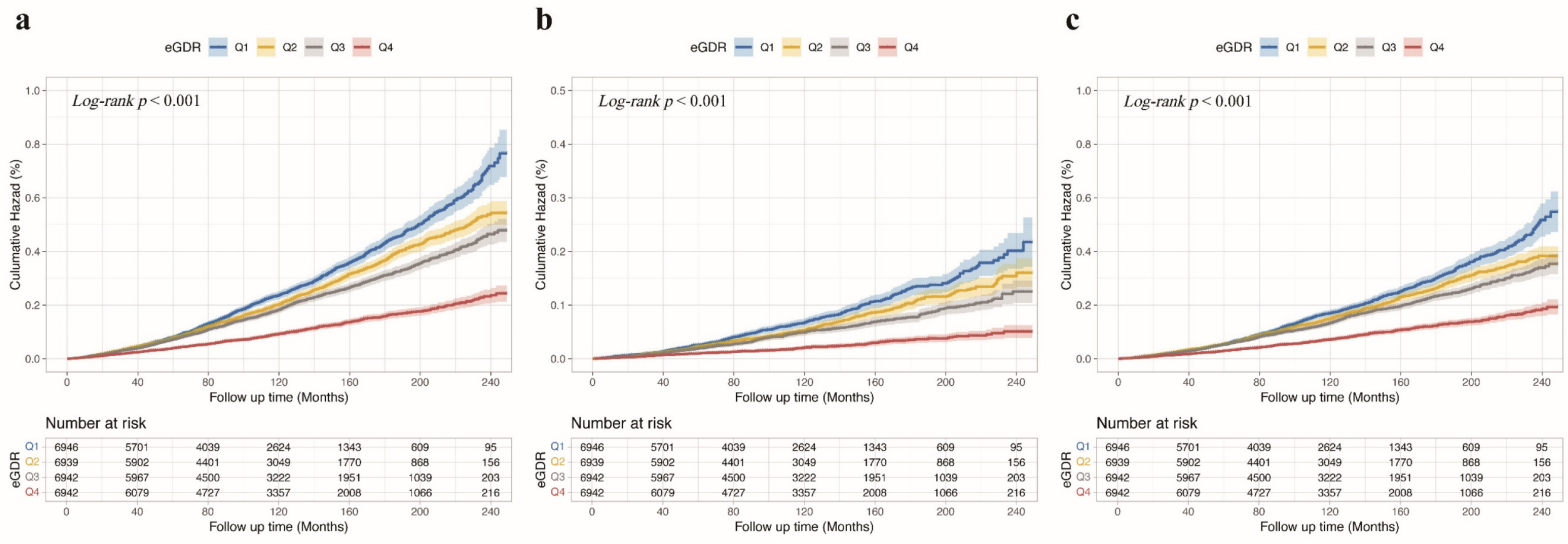
** Kaplan-Meier survival curves for death outcomes across eGDR quartiles in CKM patients. (a)** All-cause death, **(b)** cardiovascular death, **(c)** non-cardiovascular death. Kaplan-Meier curves demonstrate that higher eGDR quartiles were consistently associated with lower cumulative hazard for all-cause and cause-specific death in patients with CKM. Q1 <4.72, 4.72≤ Q2 <6.00, Q3: 6.00≤ Q3 <7.83, Q4 ≥7.83. CKM, cardiovascular-kidney-metabolic syndrome; eGDR, estimated glucose disposal rate.

**Figure 3:**
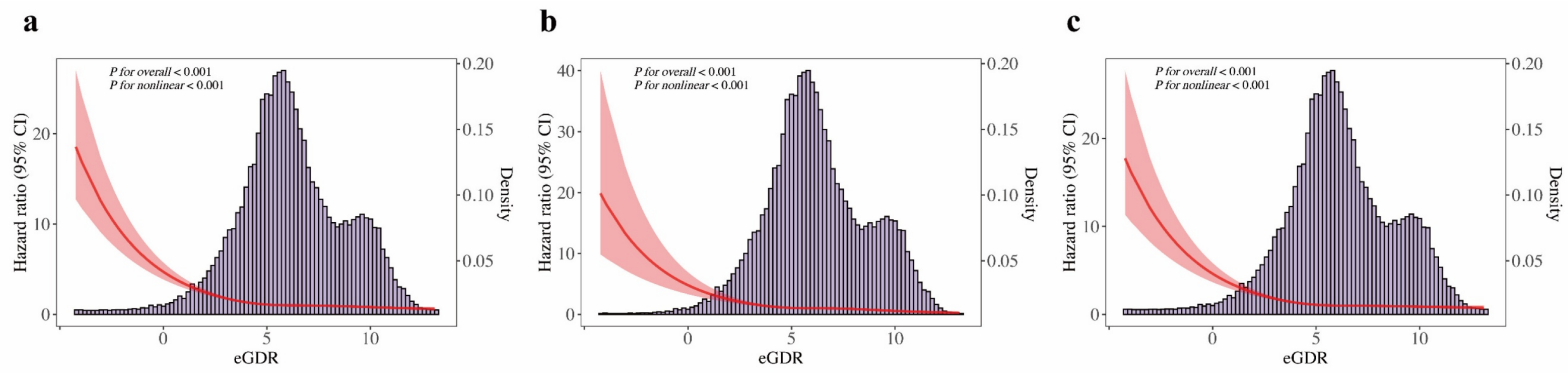
** Restricted cubic spline analyses for associations between eGDR and death outcomes in CKM patients. (a)** All-cause death, **(b)** cardiovascular death, **(c)** non-cardiovascular death. Restricted cubic spline plots show a non-linear, inverse association between eGDR and death outcomes, suggesting diminishing mortality risk with higher eGDR, especially in the lower eGDR range. CI, confidence interval; CKM, cardiovascular-kidney-metabolic syndrome; HR, hazard ratio; eGDR, estimated glucose disposal rate.

Fig. 2a shows Kaplan-Meier survival curves for all-cause death across eGDR quartiles, with significantly higher mortality in the Q1 group. Fig. 2b and Fig. 2c illustrate similar trends for cardiovascular and non-cardiovascular deaths, respectively (all *Log-rank P* < 0.001). The multivariable Cox regression analysis (Table 2) showed that each unit increase in eGDR, as a continuous variable, was linked to a 23% lower risk of all-cause death, a 31% reduction in cardiovascular death, and a 21% decrease in non-cardiovascular death, after adjusting for relevant covariates (all *P* < 0.001). Furthermore, patients in the higher quartiles of eGDR had lower risk of all-cause death (Q2: HR = 0.72, 95% CI 0.67-0.78; Q3: HR = 0.73, 95% CI 0.67-0.81; Q4: HR = 0.59, 95% CI 0.52-0.66; all *P* < 0.001), cardiovascular death (Q2: HR = 0.77, 95% CI 0.66-0.89, *P* < 0.001; Q3: HR = 0.77, 95% CI 0.64-0.92, *P* = 0.005; Q4: HR = 0.52, 95% CI 0.41-0.66, *P* < 0.001), non-cardiovascular death (Q2: HR = 0.71, 95% CI 0.65-0.78; Q3: HR = 0.72, 95% CI 0.64-0.81; Q4: HR = 0.60, 95% CI 0.53-0.69; all *P* < 0.001), compared to those in the Q1 of eGDR in CKM patients. Additionally, Fig. 3a presents a restricted cubic spline depicting a nonlinear association between eGDR and all-cause death. Fig. 3b and Fig. 3c show consistent nonlinear associations for cardiovascular and non-cardiovascular deaths (all P for overall and nonlinearity < 0.001). As eGDR levels elevated, there was a stepwise and statistically significant decrease in the risk of all-cause, cardiovascular, and non-cardiovascular death.

### Subgroup analysis

Subgroup analyses (Supplementary Fig. S3) demonstrated consistent relationships between eGDR quartiles and mortalities across different groups within the CKM patients, including age, sex, race, BMI (all *P-interaction* > 0.05). However, significant interactions were observed for CKM stage (all *P-interaction* < 0.05), with the associations being stronger in individuals with CKM stage 1/2. Nevertheless, the overall pattern of the relationship between eGDR and death outcomes remained similar.

### Sensitivity analysis

Sensitivity assessments demonstrated persistent statistical significance in eGDR-death associations following two exclusion protocols: (1) removal of 3,060 cases with documented oncological history; (2) elimination of 568 subjects experiencing death within the initial 24-month surveillance window (Supplementary Tables S6-S7). Meanwhile, the results between eGDR and death remained robust after removing the 11,690 patients with missing CRP and incorporating CRP into the multivariate Cox regression model (Supplementary Table S8).

### Predictive performance in eGDR, TyG, and HOMA-IR for death outcomes

**Figure 4:**
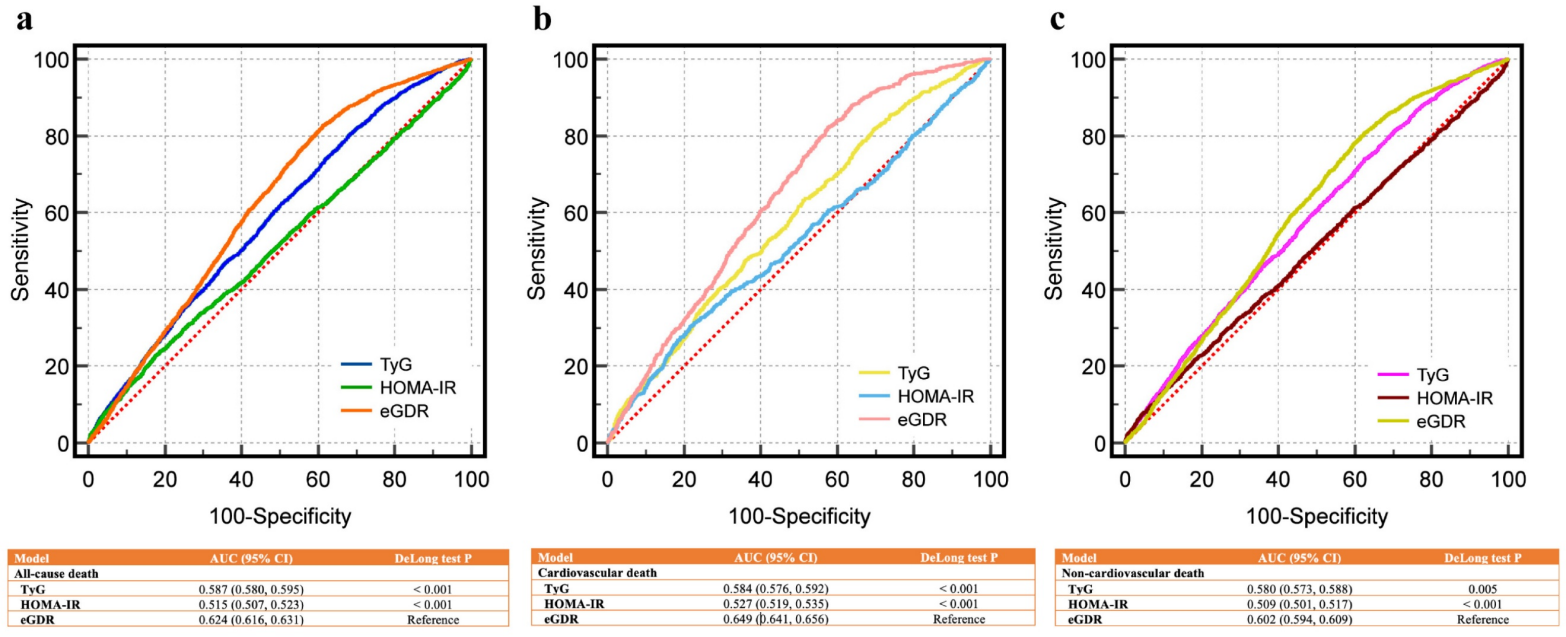
** Receiver operating characteristic curves for predicting death outcomes of CKM patients using eGDR, TyG, and HOMA-IR. (a)** All-cause death, **(b)** cardiovascular death, **(c)** non-cardiovascular death. eGDR outperformed HOMA-IR and TyG index in predicting all-cause, cardiovascular, and non-cardiovascular mortality in CKM patients, as indicated by higher AUC values and significant DeLong test results. AUC, area under curve; CI, confidence interval; CKM, cardiovascular-kidney-metabolic syndrome; eGDR, estimated glucose disposal rate; HOMA-IR, Homeostasis Model Assessment of Insulin Resistance; TyG, triglyceride-glucose index.

Fig. 4a compares the ROC curves for eGDR, TyG, and HOMA-IR in predicting all-cause death, showing superior AUC for eGDR. Fig. 4b and Fig. 4c demonstrate similar findings for cardiovascular and non-cardiovascular deaths, respectively. All comparisons were statistically significant (P < 0.01 by DeLong test). For all-cause death, the AUC of eGDR was 0.624 (95% CI: 0.616-0.631), higher than TyG (0.587) and HOMA-IR (0.515). Similar trends were observed for cardiovascular death (eGDR: 0.649 vs. TyG: 0.584 and HOMA-IR: 0.527) and non-cardiovascular death (eGDR: 0.602 vs. TyG: 0.580 and HOMA-IR: 0.509).

## Discussions

In this large, nationally representative cohort study of adults from NHANES 1999-2018, we found that lower eGDR was independently associated with a greater likelihood of advanced CKM stages and increased mortality in CKM patients. Specifically, participants with lower eGDR values had significantly higher odds of being in advanced CKM stages (stages 3-4), and lower eGDR was also linked to increased risks of all-cause, cardiovascular, and non-cardiovascular death. These associations remained robust after adjustment for multiple confounders and were further supported by sensitivity analyses. Additionally, eGDR demonstrated superior predictive performance for death outcomes compared with TyG and HOMA-IR, as evidenced by higher AUC values and statistically significant differences confirmed by the DeLong test. Our findings suggest that eGDR may serve as a valuable tool for risk stratification and death prediction in CKM populations.

The inverse relationship between eGDR and death in CKM patients supports the potential relevance of IR in systemic metabolic dysfunction. As a surrogate for insulin sensitivity, eGDR integrates visceral obesity (via WC), hypertension, and hemoglobin A1c, reflecting the synergistic effects of adiposity, chronic inflammation, and oxidative stress on cardiovascular and renal systems 20-22. While these factors have been implicated in adverse cardiometabolic outcomes, the observed associations in our study are statistical and do not establish mechanistic causality. Proposed pathways, such as IR-driven ectopic lipid accumulation, endothelial dysfunction, or fibrosis 12, remain hypothetical and warrant further mechanistic validation. Our finding that lower eGDR quartiles were associated with advanced CKM stages (3/4) may suggest clinical utility for risk stratification, but this interpretation is based on cross-sectional data. The non-linear dose-response relationship further suggests that even modest improvements in insulin sensitivity may yield significant death risk reduction, particularly in early-stage disease. Our stratified analysis revealed a stronger protective effect of higher eGDR in CKM stages 1/2 compared to advanced stages. This underscores the importance of early IR management to halt progression from subclinical metabolic dysfunction (stage 1: obesity, prediabetes) to multi-organ complications (stage 3: CKD, heart failure) 23. Notably, eGDR's predictive performance surpassed that of the TyG: a marker reliant solely on triglycerides and glucose, highlighting the added value of incorporating anthropometric and hemodynamic parameters (WC, hypertension) for holistic risk assessment. This aligns with AHA recommendations emphasizing multifactorial risk evaluation in CKM 1.

Our findings further complement the recent work by Chen *et al.* 24, which identified eGDR as a pivotal contributor to the onset and death of metabolic syndrome. eGDR demonstrates a significant association with all-cause death in diabetic patients, with its predictive power remaining independent of traditional risk factors 25. In non-diabetic individuals, reduced eGDR serves as an independent predictor of atherosclerosis, myocardial infarction, and heart failure 26. A study involving an elderly cohort found that eGDR is inversely correlated with arterial stiffness, a crucial mediator of cardiovascular events 27. Our study reveals that in patients with CKM syndrome, a lower eGDR significantly correlates with increased cardiovascular death (HR = 0.52, Q4 vs. Q1), indicating that IR directly damages the cardiovascular system by exacerbating endothelial dysfunction and myocardial fibrosis 28. The predictive value of eGDR extends beyond cardiovascular events, as low eGDR in elderly populations is significantly associated with non-cardiovascular death, including cancer and infections, potentially mediated by chronic inflammation and immune dysregulation 27, 29. This study found that in CKM patients, each standard deviation increase in eGDR was associated with a 21% lower risk of non-cardiovascular death (HR = 0.60, Q4 vs. Q1), which may be related to insulin resistance-induced systemic inflammation and frailty 30, 31.

Additionally, other recent studies have explored the relevance of eGDR in CKM populations. For instance, a prospective analysis from the China Health and Retirement Longitudinal Study reported that lower eGDR levels were associated with increased incidence of cardiovascular disease across CKM Stages 0-3, with an approximately inverse L-shaped relationship and a mediating effect of body mass index 32. Moreover, a recent investigation based on NHANES data found that lower eGDR was significantly associated with increased all-cause and cardiovascular mortality in patients with CKM, suggesting the prognostic relevance of IR in long-term outcomes 33. However, that study primarily focused on mortality risk and did not assess the association between eGDR and CKM stage classification or compare eGDR with other IR surrogates. In contrast, our study extends prior work by simultaneously evaluating the correlation between eGDR and CKM staging, its predictive utility for both all-cause and cardiovascular mortality, and its comparative performance against other commonly used insulin resistance markers.

Research demonstrates several significant merits that deserve emphasis. To begin with, the investigation employed an extensive and statistically valid sample population encompassing adult participants across the United States, with data collection adhering to standardized protocols designed to minimize potential selection bias. And rigorous adjustment for sociodemographic, lifestyle, and clinical confounders. Given the inherently protracted nature of IR progression, our longitudinal study design, featuring a median follow-up duration of 9.92 years, substantially enhances the assessment of its predictive capacity regarding mortality outcomes, including all-cause death, cardiovascular death, and non-cardiovascular death, offering critical insights into their prognostic significance. The investigation's comparative design, evaluating eGDR against standard IR markers (notably TyG and HOMA-IR), constitutes a key strength. This approach facilitates a comprehensive assessment of eGDR's predictive validity concerning mortality endpoints, contributing substantially to our knowledge of its clinical relevance. Sensitivity analyses excluding cancer patients and early deaths minimized reverse causation, while CRP-adjusted models confirmed eGDR's independence from acute inflammation.

Several limitations should be acknowledged. First, the analysis of CKM staging was based on cross-sectional data, which limits causal inference regarding disease progression. Although mortality outcomes were assessed longitudinally, the staging classification was inferred at a single time point, which constrains interpretation of progression dynamics. Second, the eGDR values were measured only once at baseline, whereas insulin resistance is known to be a dynamic process influenced by various time-varying factors. This single-timepoint measurement may weaken the validity of long-term risk predictions. Third, the eGDR formula was originally developed and validated in patients with type 1 diabetes, and its direct applicability to the CKM population requires further validation in disease-specific cohorts. Fourth, the study relied on self-reported information for several variables, including lifestyle behaviours such as smoking and alcohol consumption, which may introduce recall or reporting bias and affect risk estimates. Fifth, due to the absence of detailed pharmacological data in NHANES, we were unable to account for medication use that may influence both eGDR levels and clinical outcomes. Sixth, although extensive covariates were adjusted for, the possibility of residual or unmeasured confounding cannot be excluded. Lastly, the generalisability of our findings may be limited, as all participants were drawn from a U.S.-based population; future validation in other geographic or ethnic cohorts is needed.

## Conclusions

This research revealed a robust relationship between eGDR levels and both CKM stage and long-term mortality. While the cross-sectional nature of CKM staging limits causal inference, the consistent associations suggest that eGDR may serve as a valuable risk indicator. The findings propose that eGDR might represent a superior risk evaluation tool compared to traditional IR markers, including TyG and HOMA-IR. Future investigations should aim to confirm these results in heterogeneous populations and determine eGDR's practical implementation in clinical decision-making for CKM patients.

## Supplementary Material

Supplementary figures and tables.

## Figures and Tables

**Table 1:** Baseline characteristics categorized by quartiles of the eGDR in CKM patients

Characteristics	All (N = 27,769)	Q1 (N = 6,946)	Q2 (N = 6,939)	Q3 (N = 6,942)	Q4 (N = 6,942)	*P*
Age, years	55.00 (40.00, 67.00)	59.00 (46.00, 68.00)	60.00 (46.00, 71.00)	54.00 (40.00, 68.00)	39.00 (29.00, 53.00)	< 0.001
Male, n (%)	14,605 (52.59)	4,148 (56.65)	4,125 (56.32)	3,519 (48.07)	3,427 (46.80)	< 0.001
Race and ethnicity, n (%)						< 0.001
Non-Hispanic White	13,001 (46.82)	3,236 (46.59)	3,326 (47.93)	3,339 (48.10)	3,100 (44.66)	
Non-Hispanic Black	5,749 (20.70)	1,799 (25.90)	1,426 (20.55)	1,395 (20.10)	1,129 (16.26)	
Mexican American	4,647 (16.73)	1,114 (16.04)	1,168 (16.83)	993 (14.30)	1,372 (19.76)	
Hispanic and Others	4,372 (15.74)	797 (11.47)	1,019 (14.69)	1,215 (17.50)	1,341 (19.32)	
Body mass index, kg/m^2^	28.80 (25.40, 33.11)	35.12 (31.70, 39.72)	29.00 (26.98, 31.39)	25.20 (22.80, 28.47)	25.20 (22.37, 28.20)	< 0.001
Waist circumference, cm	100.50 (91.30, 110.70)	116.90 (111.10, 125.40)	101.70 (98.10, 105.60)	90.00 (84.20, 95.40)	89.50 (80.70, 97.30)	< 0.001
Systolic blood pressure, mmHg	126.00 (116.00, 139.00)	132.00 (122.00, 143.00)	132.00 (122.00, 144.00)	128.00 (117.00, 140.00)	112.00 (105.00, 119.00)	
Diastolic blood pressure, mmHg	73.00 (64.00, 81.00)	75.00 (65.00, 83.00)	75.00 (66.00, 83.00)	74.00 (66.00, 82.00)	67.00 (61.00, 73.00)	
Poverty income ratio	2.20 (1.17, 4.12)	2.04 (1.12, 3.84)	2.26 (1.21, 4.18)	2.24 (1.20, 4.21)	2.33 (1.18, 4.38)	< 0.001
Education, n (%)						< 0.001
Less than high school	3,327 (11.98)	862 (12.41)	984 (14.18)	776 (11.18)	705 (10.16)	
High school or equivalent	10,713 (38.58)	2,853 (41.07)	2,687 (38.72)	2,685 (38.68)	2,488 (35.84)	
College or above	13,729 (49.44)	3,231 (46.52)	3,268 (47.10)	3,481 (50.14)	3,749 (54.00)	
Marital status, n (%)						< 0.001
Unmarried	3,714 (13.37)	812 (11.69)	634 (9.14)	905 (13.04)	1,363 (19.63)	
Married	17,163 (61.81)	4,226 (60.84)	4,487 (64.66)	4,155 (59.85)	4,295 (61.87)	
Divorcee	6,892 (24.82)	1,908 (27.47)	1,818 (26.20)	1,882 (27.11)	1,284 (18.50)	
Smoking status, n (%)						< 0.001
Never smoker	14,314 (51.55)	3,339 (48.07)	3,519 (50.71)	3,645 (52.51)	3,811 (54.90)	
Former smoker	7,876 (28.36)	2,438 (35.10)	2,177 (31.37)	1,749 (25.19)	1,512 (21.78)	
Current smoker	5,579 (20.09)	1,169 (16.83)	1,243 (17.91)	1,548 (22.30)	1,619 (23.32)	
Alcohol consumption, n (%)						< 0.001
Non-drinker	17,534 (63.14)	4,658 (67.06)	4,519 (65.12)	4,399 (63.37)	3,958 (57.02)	
Mild to moderate	6,375 (22.96)	1,455 (20.95)	1,532 (22.08)	1,583 (22.80)	1,805 (26.00)	
Heavy	6,375 (22.96)	833 (11.99)	888 (12.80)	960 (13.83)	1,179 (16.98)	
Physical activity, n (%)						< 0.001
Less than moderate	16,258 (58.55)	4,252 (61.22)	4,053 (58.41)	4,045 (58.27)	3,908 (56.30)	
Moderate	6,906 (24.87)	1,719 (24.75)	1,776 (25.59)	1,732 (24.95)	1,679 (24.19)	
Vigorous	4,605 (16.58)	975 (14.04)	1,110 (16.00)	1,165 (16.78)	1,355 (19.52)	
Laboratory indicators						
Hemoglobin A1c, %	5.60 (5.30, 6.00)	6.00 (5.60, 7.00)	5.60 (5.30, 5.90)	5.40 (5.20, 5.70)	5.30 (5.10, 5.60)	< 0.001
Total Cholesterol, mg/dL	195.00 (169.00, 224.00)	191.00 (164.00, 221.00)	200.00 (173.00, 229.00)	198.00 (172.00, 225.00)	189.00 (165.00, 216.00)	< 0.001
HDL-C, mg/dL	50.00 (41.00, 61.00)	44.00 (38.00, 53.00)	48.00 (40.00, 59.00)	54.00 (44.00, 67.00)	55.00 (46.00, 66.00)	< 0.001
eGFR, ml/min/1.73m^2^	91.20 (74.32, 105.98)	87.98 (69.85, 102.54)	86.41 (70.35, 100.57)	91.32 (75.39, 105.37)	103.08 (87.95, 116.38)	< 0.001
UACR, mg/g	7.67 (4.72, 16.76)	10.00 (5.59, 26.94)	7.85 (4.86, 16.52)	7.33 (4.66, 14.25)	5.95 (4.05, 10.68)	< 0.001
10-year CVD risk score	5.71 (1.55, 15.09)	10.70 (4.08, 19.47)	7.96 (2.79, 17.77)	4.60 (1.32, 13.22)	1.08 (0.40, 3.60)	< 0.001
CKM Stage, n (%)						< 0.001
CKM Stage 1	3,134 (11.29)	7 (0.10)	31 (0.45)	229 (3.30)	2,867 (41.30)	
CKM Stage 2	17,878 (64.38)	4,550 (65.51)	4,887 (70.43)	5,160 (74.33)	3,281 (47.26)	
CKM Stage 3	3,130 (11.27)	1,052 (15.15)	981 (14.14)	775 (11.16)	322 (4.64)	
CKM Stage 4	3,627 (13.06)	1,337 (19.25)	1,040 (14.99)	778 (11.21)	472 (6.80)	

eGDR: Q1 < 4.72, 4.72 ≤ Q2 < 6.00, Q3: 6.00 ≤ Q3 < 7.83, Q4 ≥ 7.83. Abbreviations: CKM, cardiovascular-kidney-metabolic syndrome; CVD, cardiovascular disease; eGDR, estimated glucose disposal rate; eGFR, estimated glomerular filtration rate; HDL-C, high-density lipoprotein cholesterol; SII, systemic immune-inflammation index; UACR, urinary albumin to creatinine ratio.

**Table 2:** The interaction between eGDR and mortality outcomes in CKM patients

	Model I		Model II		Model III	
	HR (95% CI)	*P*	HR (95% CI)	*P*	HR (95% CI)	*P*
**All-cause death**						
Continues eGDR	0.72 (0.70, 0.74)	< 0.001	0.74 (0.70, 0.78)	< 0.001	0.77 (0.73, 0.80)	< 0.001
eGDR quartiles						
Q1	*Reference*		*Reference*		*Reference*	
Q2	0.86 (0.80, 0.92)	< 0.001	0.67 (0.62, 0.73)	< 0.001	0.72 (0.67, 0.78)	< 0.001
Q3	0.73 (0.68, 0.78)	< 0.001	0.68 (0.62, 0.75)	< 0.001	0.73 (0.67, 0.81)	< 0.001
Q4	0.28 (0.25, 0.30)	< 0.001	0.54 (0.48, 0.61)	< 0.001	0.59 (0.52, 0.66)	< 0.001
**Cardiovascular death**						
Continues eGDR	0.65 (0.62, 0.69)	< 0.001	0.66 (0.60, 0.73)	< 0.001	0.69 (0.63, 0.76)	< 0.001
eGDR quartiles						
Q1	*Reference*		*Reference*		*Reference*	
Q2	0.83 (0.72, 0.94)	0.005	0.72 (0.62, 0.83)	< 0.001	0.77 (0.66, 0.89)	< 0.001
Q3	0.64 (0.56, 0.74)	< 0.001	0.72 (0.60, 0.87)	< 0.001	0.77 (0.64, 0.92)	0.005
Q4	0.20 (0.16, 0.24)	< 0.001	0.48 (0.38, 0.61)	< 0.001	0.52 (0.41, 0.66)	< 0.001
**Non cardiovascular death**						
Continues eGDR	0.75 (0.72, 0.77)	< 0.001	0.77 (0.72, 0.81)	< 0.001	0.79 (0.75, 0.84)	< 0.001
eGDR quartiles						
Q1	*Reference*		*Reference*		*Reference*	
Q2	0.87 (0.80, 0.95)	0.001	0.66 (0.60, 0.73)	< 0.001	0.71 (0.65, 0.78)	< 0.001
Q3	0.76 (0.70, 0.83)	< 0.001	0.66 (0.59, 0.75)	< 0.001	0.72 (0.64, 0.81)	< 0.001
Q4	0.31 (0.28, 0.35)	< 0.001	0.56 (0.49, 0.64)	< 0.001	0.60 (0.53, 0.69)	< 0.001

eGDR: Q1 < 4.72, 4.72 ≤ Q2 < 6.00, Q3: 6.00 ≤ Q3 < 7.83, Q4 ≥ 7.83. Model I: Unadjusted; Model II: Adjusted age, sex, race and ethnicity, body mass index; Model III: Based on Model II further adjusted poverty income ratio, marital states, education, smoking status, alcohol consumption, physical activity. Abbreviations: CI, confidence interval; CKM, cardiovascular-kidney-metabolic syndrome; eGDR, estimated glucose disposal rate; HR, hazard ratio.

## Abbreviations

AHA: American Heart Association
CKM: Cardiovascular-kidney-metabolism syndrome
CKD: Chronic kidney disease
CRP: C-reactive protein
CVD: Cardiovascular disease
eGDR: estimated glucose disposal rate
HOMA-IR: Homeostasis Model Assessment for Insulin Resistance
PIR: Poverty income ratio
T2DM: Type 2 diabetes mellitus
TyG: Triglyceride-glucose index
