# Supplementary material for: Role of Estimated Glucose Disposal Rate in Staging and Death Risk of Cardiovascular-Kidney-Metabolic Syndrome: Insights from NHANES 1999-2018
Source: Int J Med Sci. 2025 Aug 16;22(14):3779–88. doi: 10.7150/ijms.116708 (PMC12434822; doi:10.7150/ijms.116708)
Supplement: Supplementary file 1 — Supplementary figures and tables. [file ijmsv22p3779s1.pdf]

Supplementary Table S1. Checklist for Strengthening the Reporting of Observational Studies in Epidemiology (STROBE) for this cohort study

	Item No	Recommendation	Page No
**Title and abstract**	1	(*a*) Indicate the study's design with a commonly used term in the title or the abstract	1,3
		(*b*) Provide in the abstract an informative and balanced summary of what was done and what was found	3,4
**Introduction**			
Background/rationale	2	Explain the scientific background and rationale for the investigation being reported	5,6
Objectives	3	State specific objectives, including any prespecified hypotheses	6
**Methods**			
Study design	4	Present key elements of study design early in the paper	7,8
Setting	5	Describe the setting, locations, and relevant dates, including periods of recruitment, exposure, follow-up, and data collection	7,8
Participants	6	(*a*) Give the eligibility criteria, and the sources and methods of selection of participants. Describe methods of follow-up	7,8
		(*b*) For matched studies, give matching criteria and number of exposed and unexposed	N/A
Variables	7	Clearly define all outcomes, exposures, predictors, potential confounders, and effect modifiers. Give diagnostic criteria, if applicable	7,8,9
Data sources/ measurement	8*	For each variable of interest, give sources of data and details of methods of assessment (measurement). Describe comparability of assessment methods if there is more than one group	7
Bias	9	Describe any efforts to address potential sources of bias	9,10
Study size	10	Explain how the study size was arrived at	7
Quantitative variables	11	Explain how quantitative variables were handled in the analyses. If applicable, describe which groupings were chosen and why	7
Statistical methods	12	(*a*) Describe all statistical methods, including those used to control for confounding	9,10,11
		(*b*) Describe any methods used to examine subgroups and interactions	10

		(*c*) Explain how missing data were addressed	7
		(*d*) If applicable, explain how loss to follow-up was addressed	7
		(*e*) Describe any sensitivity analyses	10
**Results**			
Participants	13*	(a) Report numbers of individuals at each stage of study—eg numbers potentially eligible, examined for eligibility, confirmed eligible, included in the study, completing follow-up, and analysed	12,13
		(b) Give reasons for non-participation at each stage	7
		(c) Consider use of a flow diagram	Supplementary Fig. S1
Descriptive data	14*	(a) Give characteristics of study participants (eg demographic, clinical, social) and information on exposures and potential confounders	12
		(b) Indicate number of participants with missing data for each variable of interest	7
		(c) Summarise follow-up time (eg, average and total amount)	12
Outcome data	15*	Report numbers of outcome events or summary measures over time	12
Main results	16	(*a*) Give unadjusted estimates and, if applicable, confounder-adjusted estimates and their precision (eg, 95% confidence interval). Make clear which confounders were adjusted for and why they were included	12,13,14
		(*b*) Report category boundaries when continuous variables were categorized	12,13,14
		(*c*) If relevant, consider translating estimates of relative risk into absolute risk for a meaningful time period	12,13,14
Other analyses	17	Report other analyses done-eg analyses of subgroups and interactions, and sensitivity analyses	14,15
**Discussion**			
Key results	18	Summarise key results with reference to study objectives	16
Limitations	19	Discuss limitations of the study, taking into account sources of potential bias or imprecision. Discuss both direction and magnitude of any potential bias	19
Interpretation	20	Give a cautious overall interpretation of results considering objectives, limitations, multiplicity of analyses, results from similar studies, and other relevant evidence	16,17,18

Generalisability	21	Discuss the generalisability (external validity) of the study results	16,17,18,19
**Other information**			
Funding	22	Give the source of funding and the role of the funders for the present study and, if applicable, for the original study on which the present article is based	21

*Give information separately for exposed and unexposed groups

Note: An Explanation and Elaboration article discusses each checklist item and gives methodological background and published examples of transparent reporting. The STROBE checklist is best used in conjunction with this article (freely available on the Web sites of PLoS Medicine at http://www.plosmedicine.org/, Annals of Internal Medicine at http://www.annals.org/, and Epidemiology at http://www.epidem.com/). Information on the STROBE Initiative is available at http://www.strobe-statement.org

Supplementary Table S2. Definitions of CKM

CKM conditions	Definition	CKM indicators	Threshold for CKM indicators
CVD	Individuals with clinical CVD or subclinical CVD	Clinical CVD	History of chronic heart failure, coronary heart disease, heart attack, or stroke
		Subclinical CVD	Any of the following criterion is met:<br>1) Very high-risk CKD in KDIGO classification: UACR ≥ 300 mg/g and eGFR ≤ 45-59 ml/min/1.73m2, UACR ≥ 30 mg/g and eGFR ≤ 30-44 ml/min/1.73m2, or eGFR ≤ 29 ml/min/1.73m2.<br>2) Predicted 10-year CVD risk ≥ 20%
Kidney diseases	Individuals with CKD	CKD	Moderate-to-high-risk CKD in KDIGO classification: UACR ≥ 30 mg/g and eGFR ≥ 60 ml/min/1.73m2, UACR < 300 mg/g and eGFR ≤ 45-59 ml/min/1.73m2, or UACR < 30 mg/g and eGFR ≤ 30-44 ml/min/1.73m2.
Metabolic disorders	Individuals with overweight/obesity, abdominal obesity, prediabetes, diabetes, hypertension, hypertriglyceridemia or MetS	Overweight/obesity	BMI ≥25 kg/m2 (or ≥23 kg/m2 if Asian ancestry) *
		Abdominal obesity	Waist circumference ≥88/102 cm in female/male (or if Asian ancestry ≥80/90 cm in female/male)
		Prediabetes	Fasting blood glucose ≥ 100-124 mg/dL or HbA1c ≥ 5.7%-6.4% and without self-reported diagnosis of diabetes, use of insulin, or oral hypoglycemic agents
		Diabetes	Fasting blood glucose ≥ 125 mg/dL or HbA1c ≥ 6.5% or self-reported diagnosis of diabetes, use of insulin, or oral hypoglycemic agents
		Hypertension	SBP ≥130 mm Hg or DBP ≥80 mm Hg or self-reported diagnosis of hypertension or use of antihypertensive medications
		Hypertriglyceridemia	Triglycerides ≥ 135 mg/dL
		MetS	MetS is defined by the presence of 3 or more of the following:<br>1) Waist circumference ≥88/102 cm in female/male (or if Asian ancestry ≥80/90 cm in female/male).<br>2) HDL cholesterol <50/40 mg/dL in female/male.

	3) Triglycerides ≥150 mg/dL.<br>4) Elevated blood pressure (SBP ≥130 mm Hg or DBP ≥80 mm Hg and/or use of antihypertensive medications)<br>5) Fasting blood glucose ≥100 mg/dL

*Asian was not listed as a separate race/ethnicity until NAHNES 2011-2012, therefore the uniform threshold for BMI and waist circumference was used in all participants in NHANES 1999-2010.

Abbreviations: BMI, body mass index; CKD, chronic kidney disease; CKM, cardiovascular-kidney-metabolic syndrom; CVD, cardiovascular disease; DBP, diastolic blood pressure; eGFR, estimated glomerular filtration rate; HDL-C, high-density lipoprotein cholesterol; KDIGO, The Kidney Disease: Improving Global Outcomes; MetS, metabolic syndrome; SBP, systolic blood pressure; UACR, urinary albumin to creatinine ratio.

Supplementary Table S3. Detailed algorithm of the simplified 10-year cardiovascular disease risk models

Sex	Calculation
Women	log-Odds = -3.307728 + 0.7939329 × (age – 55) /10 + 0.0305239 × ((TC – HDL-C) × 0.02586 – 3.5) – 0.1606857 × (HDL-C – 1.3) /0.3 – 0.2394003 × (min(SBP, 110) – 110) /20 + 0.360078 × (max(SBP, 110) – 130) /20 + 0.8667604 × (if diabetes) + 0.5360739 × (if current smoker) + 0.6045917 × (min(eGFR, 60) – 60) / -15 + 0.0433769 × (max(eGFR, 60) – 90) / -15 + 0.3151672 × (if using anti-hypertensive medication) – 0.1477655 × (if using statin) – 0.0663612 × (if using anti-hypertensive medication) × (max(SBP, 110) – 130) /20 + 0.1197879 × (if using statin) × (TC – HDL-C – 3.5) – 0.0819715 × (age – 55) /10 × (TC – HDL-C – 3.5) + 0.0306769 × (age – 55) /10 × (HDL-C – 1.3) /0.3 – 0.0946348 × (age – 55) /10 × (max(SBP, 110) – 130) /20 – 0.27057 × (age – 55) /10 × (if diabetes) – 0.078715 × (age – 55) /10 × (if current smoker) – 0.1637806 × (age – 55) /10 × (min(eGFR, 60) – 60) / -15<br>Risk = exp(log-Odds) / (1 + exp(log-Odds))
Men	log-Odds = -3.031168 + 0.7688528 × (age – 55) /10 + 0.0736174 × ((TC – HDL-C) × 0.02586 – 3.5) – 0.0954431 × (HDL-C – 1.3) /0.3 – 0.4347345 × (min(SBP, 110) – 110) /20 + 0.3362658 × (max(SBP, 110) – 130) /20 + 0.7692857 × (if diabetes) + 0.4386871 × (if current smoker) + 0.5378979 × (min(eGFR, 60) – 60) / -15 + 0.0164827 × (max(eGFR, 60) – 90) / -15 + 0.288879 × (if using anti-hypertensive medication) – 0.1337349 × (if using statin) – 0.0475924 × (if using anti-hypertensive medication) × (max(SBP, 110) – 130) /20 + 0.150273 × (if using statin) × (TC – HDL-C – 3.5) – 0.0517874 × (age – 55) /10 × (TC – HDL-C – 3.5) + 0.0191169 × (age – 55) /10 × (HDL-C – 1.3) /0.3 – 0.1049477 × (age – 55) /10 × (max(SBP, 110) – 130) /20 – 0.2251948 × (age – 55) /10 × (if diabetes) – 0.0895067 × (age – 55) /10 × (if current smoker) – 0.1543702 × (age – 55) /10 × (min(eGFR, 60) – 60) / -15<br>Risk = exp(log-Odds) / (1 + exp(log-Odds))

Abbreviations: eGFR, estimated glomerular filtration rate; HDL-C, high-density lipoprotein cholesterol; SBP, systolic blood pressure; TC, total cholesterol

Supplementary Table S4. Detailed algorithm for evaluating each CKM stage

CKM stages	Definition	Criterion	Threshold for CKM conditions
Stage 0: No CKM risk factors	Individuals with normal BMI and waist circumference, normoglycemia, normotension, a normal lipid profile, and no evidence of CKD or subclinical or clinical CVD	All criteria are met	BMI <25 kg/m^2 (or <23 kg/m^2 if Asian ancestry)*
			Waist circumference <88/102 cm in female/male (or if Asian ancestry <80/90 cm in female/male)
			Fasting blood glucose < 100 mg/dL and HbA1c < 5.7% and without self-reported diagnosis of diabetes, use of insulin, or oral hypoglycemic agents
			SBP <130 mm Hg and DBP <80 mm Hg without self-reported diagnosis of hypertension or use of antihypertensive medications
			HDL cholesterol >50/40 mg/dL in female/male and triglycerides < 150 mg/dL
			Low-risk CKD in KDIGO classification according to eGFR and UACR: UACR < 30 mg/g and eGFR ≥ 60 ml/min/1.73m^2.
			Predicted 10-year CVD risk < 20%
			No clinical CVD
Stage 1: Excess or dysfunctional adiposity	Individuals with overweight/obesity, abdominal obesity, or dysfunctional adipose tissue, without the presence of other metabolic risk factors or CKD	Any of the three criteria is met	Overweight/obesity
			Abdominal obesity
			Prediabetes
		All criteria are met	SBP <130 mm Hg and DBP <80 mm Hg without self-reported diagnosis of hypertension or use of antihypertensive medications
			HDL cholesterol >50/40 mg/dL in female/male and triglycerides <150 mg/dL
			Low-risk CKD in KDIGO classification according to eGFR and UACR: UACR < 30 mg/g and eGFR ≥ 60 ml/min/1.73m^2
			Predicted 10-year CVD risk < 20%
			No clinical CVD
Stage 2: Metabolic risk factors and CKD	Individuals with metabolic risk factors (hypertriglyceridemia, hypertension, MetS, diabetes), or CKD	Any of the five criteria is met	Hypertriglyceridemia
			Hypertension
			diabetes
			MetS
			Moderate-to-high-risk CKD in KDIGO classification
		All criteria are met	No very high-risk CKD in KDIGO classification
			Predicted 10-year CVD risk < 20%

			No clinical CVD
Stage 3: Subclinical CVD in CKM	Subclinical CVD among individuals with excess/dysfunctional adiposity, other metabolic risk factors, or CKD	Any of the two criteria is met	Very high-risk CKD in KDIGO classification<br>Predicted 10-year CVD risk ≥ 20%
		Any of the eight criteria is met	Overweight/obesity<br>Abdominal obesity<br>Prediabetes<br>Hypertriglyceridemia<br>Hypertension<br>diabetes<br>MetS<br>Moderate-to-high-risk CKD in KDIGO classification
		The criterion is met	No clinical CVD
Stage 4: Clinical CVD in CKM	Clinical CVD among individuals with excess/dysfunctional adiposity, other metabolic risk factors, or CKD	The criterion is met	Clinical CVD
		Any of the nine criteria is met	Overweight/obesity<br>Abdominal obesity<br>Prediabetes<br>Hypertriglyceridemia<br>Hypertension<br>diabetes<br>MetS<br>Moderate-to-high-risk CKD in KDIGO classification<br>Very high-risk CKD in KDIGO classification

*Asian was not listed as a separate race/ethnicity until NHANES 2011-2012, therefore the uniform threshold for BMI and waist circumference was used in all participants in NHANES 1999-2010.

Abbreviations: BMI, body mass index; CKD, chronic kidney disease; CKM, cardiovascular-kidney-metabolic syndrom; CVD, cardiovascular disease; DBP, diastolic blood pressure; eGFR, estimated glomerular filtration rate; HDL-C, high-density lipoprotein; KDIGO, The Kidney Disease: Improving Global Outcomes; MetS, metabolic syndrome; SBP, systolic blood pressure; UACR, urinary albumin to creatinine ratio.

Supplementary Table S5. Baseline characteristics categorised by CKM stages in all participants

Characteristics	All (n = 29,290)	Non-CKM (n = 1,521)	CKM Stage 1 (n = 3,134)	CKM Stage 2 (n =17,878)	CKM Stage 3 (n = 3,130)	CKM Stage 4 (n = 3,627)	***P***
Age, years	53.00 (39.00, 66.00)	31.00 (25.00, 42.00)	40.00 (30.00, 51.00)	50.00 (38.00, 61.00)	78.00 (72.00, 80.00)	68.00 (60.00, 77.00)	< 0.001
Male, n (%)	15,219.00 (51.96%)	614.00 (40.37%)	1,542.00 (49.20%)	9,072.00 (50.74%)	1,807.00 (57.73%)	2,184.00 (60.22%)	< 0.001
Race, n (%)							< 0.001
Non-Hispanic White	13,818.00 (47.18%)	614.00 (40.37%)	1,267.00 (40.43%)	7,719.00 (43.18%)	1,888.00 (60.32%)	2,127.00 (58.64%)	
Non-Hispanic Black	5,984.00 (20.43%)	235.00 (15.45%)	629.00 (20.07%)	3,869.00 (21.64%)	530.00 (16.93%)	721.00 (19.88%)	
Mexican American	4,844.00 (16.54%)	197.00 (12.95%)	607.00 (19.37%)	3,253.00 (18.20%)	404.00 (12.91%)	383.00 (10.56%)	
Hispanic and Others	4,644.00 (15.86%)	272.00 (17.88%)	631.00 (20.13%)	3,037.00 (16.99%)	308.00 (9.84%)	396.00 (10.92%)	
Body mass index, kg/m^2	28.40 (24.79, 32.80)	22.00 (20.39, 23.40)	27.31 (25.13, 30.70)	29.20 (25.55, 33.70)	27.98 (24.80, 31.56)	29.10 (25.66, 33.50)	< 0.001
Waist circumference, cm	99.50 (89.60, 110.00)	78.70 (74.10, 83.30)	94.15 (87.50, 102.70)	100.60 (91.10, 111.10)	102.20 (93.60, 111.00)	104.20 (95.50, 114.50)	< 0.001
Systolic blood pressure, mmHg	125.00 (114.00, 138.00)	108.00 (102.00, 115.00)	113.00 (106.00, 119.00)	127.00 (117.00, 138.00)	139.00 (125.00, 155.00)	130.00 (117.00, 145.00)	< 0.001
Diastolic blood pressure, mmHg	72.00 (64.00, 80.00)	66.00 (60.00, 71.00)	68.00 (62.00, 73.00)	76.00 (68.00, 83.00)	66.00 (58.00, 75.00)	68.00 (59.00, 77.00)	< 0.001
Poverty income ratio	2.22 (1.18, 4.14)	2.75 (1.32, 4.84)	2.75 (1.32, 4.84)	2.75 (1.32, 4.84)	1.96 (1.20, 3.35)	1.81 (1.08, 3.37)	< 0.001
Education, n (%)							< 0.001
Less than high school	3,399.00 (11.60%)	72.00 (4.73%)	243.00 (7.75%)	1,880.00 (10.52%)	638.00 (20.38%)	566.00 (15.61%)	
High school or equivalent	11,154.00 (38.08%)	441.00 (28.99%)	1,021.00 (32.58%)	6,868.00 (38.42%)	1,243.00 (39.71%)	1,581.00 (43.59%)	
College or above	14,737.00 (50.31%)	1,008.00 (66.27%)	1,870.00 (59.67%)	9,130.00 (51.07%)	1,249.00 (39.90%)	1,480.00 (40.81%)	
Marital status, n (%)							< 0.001
Unmarried	4,258.00 (14.54%)	544.00 (35.77%)	727.00 (23.20%)	2,650.00 (14.82%)	114.00 (3.64%)	223.00 (6.15%)	
Married	17,991.00 (61.42%)	828.00 (54.44%)	1,961.00 (62.57%)	11,324.00 (63.34%)	1,757.00 (56.13%)	2,121.00 (58.48%)	
Divorcee	7,041.00 (24.04%)	149.00 (9.80%)	446.00 (14.23%)	3,904.00 (21.84%)	1,259.00 (40.22%)	1,283.00 (35.37%)	
Smoking status, n (%)							< 0.001
Never smoker	15,260.00 (52.10%)	946.00 (62.20%)	1,912.00 (61.01%)	9,489.00 (53.08%)	1,544.00 (49.33%)	1,369.00 (37.74%)	
Former smoker	8,102.00 (27.66%)	946.00 (62.20%)	649.00 (20.71%)	4,335.00 (24.25%)	1,365.00 (43.61%)	1,527.00 (42.10%)	

Current smoker	5,928.00 (20.24%)	349.00 (22.95%)	573.00 (18.28%)	4,054.00 (22.68%)	221.00 (7.06%)	731.00 (20.15%)	
Alcohol consumption, n (%)							< 0.001
Non-drinker	18,282.00 (62.42%)	748.00 (49.18%)	1,645.00 (52.49%)	10,422.00 (58.30%)	2,662.00 (85.05%)	2,805.00 (77.34%)	
Mild to moderate	6,864.00 (23.43%)	489.00 (32.15%)	939.00 (29.96%)	4,537.00 (25.38%)	361.00 (11.53%)	538.00 (14.83%)	
Heavy	4,144.00 (14.15%)	284.00 (18.67%)	550.00 (17.55%)	2,919.00 (16.33%)	107.00 (3.42%)	538.00 (14.83%)	
Physical activity, n (%)							< 0.001
Less than moderate	17,067.00 (58.27%)	809.00 (53.19%)	1,716.00 (54.75%)	10,174.00 (56.91%)	2,038.00 (65.11%)	2,330.00 (64.24%)	
Moderate	7,265.00 (24.80%)	359.00 (23.60%)	741.00 (23.64%)	4,405.00 (24.64%)	836.00 (26.71%)	924.00 (25.48%)	
Vigorous	4,958.00 (16.93%)	353.00 (23.21%)	677.00 (21.60%)	3,299.00 (18.45%)	256.00 (8.18%)	373.00 (10.28%)	
Laboratory indicators							
Hemoglobin A1c, %	5.50 (5.30, 5.90)	5.20 (5.00, 5.30)	5.40 (5.10, 5.60)	5.40 (5.10, 5.60)	5.80 (5.50, 6.60)	5.80 (5.50, 6.40)	< 0.001
Total Cholesterol, mg/dL	194.00 (168.00, 223.00)	178.00 (157.00, 201.00)	191.00 (166.00, 214.00)	200.00 (174.00, 229.00)	190.00 (164.00, 217.00)	179.00 (152.00, 210.00)	< 0.001
HDL-C, mg/dL	50.00 (41.00, 61.00)	62.00 (54.00, 72.00)	57.00 (51.00, 66.00)	48.00 (40.00, 60.00)	49.00 (41.00, 60.00)	47.00 (39.00, 57.00)	< 0.001
eGFR, ml/min/1.73m^2	92.06 (75.30, 107.04)	107.97 (95.66, 120.28)	104.16 (90.92, 116.67)	95.51 (81.61, 108.54)	64.49 (50.31, 79.32)	73.39 (55.82, 88.75)	< 0.001
UACR, mg/g	7.50 (4.67, 16.04)	5.68 (4.00, 8.75)	5.14 (3.74, 7.74)	7.23 (4.64, 14.46)	14.53 (7.27, 41.18)	11.44 (6.15, 34.00)	< 0.001
Multidimensional score							
SII	472.50 (336.41, 663.92)	409.79 (299.38, 581.04)	431.88 (312.00, 598.54)	473.59 (339.68, 660.00)	502.68 (355.76, 718.26)	506.32 (345.63, 724.29)	< 0.001
10-year CVD risk score	5.08 (1.29, 14.39)	0.47 (0.23, 1.10)	1.02 (0.41, 2.78)	4.07 (1.37, 9.53)	25.96 (22.67, 30.61)	18.26 (9.28, 27.38)	< 0.001
Life's Simple 7 score	8.00 (6.00, 9.00)	11.00 (10.00, 12.00)	9.00 (8.00, 11.00)	7.00 (6.00, 9.00)	7.00 (5.00, 8.00)	6.00 (5.00, 8.00)	< 0.001
Frailty score	0.13 (0.08, 0.20)	0.08 (0.05, 0.11)	0.09 (0.06, 0.13)	0.13 (0.08, 0.18)	0.17 (0.12, 0.23)	0.25 (0.18, 0.34)	< 0.001
HEI-2015	50.30 (41.11, 60.10)	50.49 (41.37, 60.93)	49.99 (41.32, 60.03)	49.71 (40.50, 59.37)	53.43 (44.37, 63.08)	50.61 (41.33, 60.60)	< 0.001
eGDR	6.15 (4.80, 8.36)	11.24 (10.82, 11.65)	9.73 (8.89, 10.39)	5.88 (4.69, 7.22)	5.42 (4.27, 6.55)	5.32 (4.04, 6.57)	< 0.001

eGDR: Q1 < 4.80, 4.80 ≤ Q2 < 6.15, Q3: 6.15 ≤ Q3 < 8.36, Q4 ≥ 8.36.

Abbreviations: CKM, cardiovascular-kidney-metabolic syndrome; CVD, cardiovascular disease; eGDR, estimated glucose disposal rate; eGFR, estimated glomerular filtration rate; HDL-C, high-density lipoprotein cholesterol; SII, systemic immune-inflammation index; UACR, urinary albumin to creatinine ratio; HEI, Healthy Eating Index.

Supplementary Table S6. Sensitivity analysis of eGDR and mortality outcomes in CKM patients without history of cancer

**eGDR quartiles**	**All-cause death**		**Cardiovascular death**		**Non-cardiovascular death**	
	**HR (95% CI)**	***P***	**HR (95% CI)**	***P***	**HR (95% CI)**	***P***
Continues eGDR	*0.79 (0.74, 0.84)*	< 0.001	*0.73 (0.65, 0.83)*	< 0.001	*0.81 (0.75, 0.87)*	< 0.001
eGDR quartiles						
Q1	*Reference*		*Reference*		*Reference*	
Q2	0.69 (0.63 0.76)	< 0.001	0.76 (0.64, 0.90)	< 0.001	0.67 (0.60 0.75)	< 0.001
Q3	0.70 (0.63, 0.78)	< 0.001	0.76 (0.62, 0.93)	0.009	0.68 (0.59, 0.77)	< 0.001
Q4	0.59 (0.52, 0.67)	< 0.001	0.58 (0.45, 0.74)	< 0.001	0.58 (0.50, 0.68)	< 0.001

GDR: Q1 < 4.74, 4.74 ≤ Q2 < 6.05, Q3: 6.05 ≤ Q3 < 7.96, Q4 ≥ 7.96.

Models were adjusted for age, sex, race and ethnicity, body mass index, waist circumference, poverty income ratio, marital states, education, smoking status, alcohol consumption, physical activity.

Abbreviations: CI, confidence interval; CKM, cardiovascular-kidney-metabolic syndrome; eGDR, estimated glucose disposal rate; HR, hazard ratio.

Supplementary Table S7. Sensitivity analysis of eGDR and mortality outcomes in CKM patients not dead within 2 years

**eGDR quartiles**	**All-cause death**		**Cardiovascular death**		**Non-cardiovascular death**	
	**HR (95% CI)**	***P***	**HR (95% CI)**	***P***	**HR (95% CI)**	***P***
Continues eGDR	0.75 (0.72, 0.79)	< 0.001	0.67 (0.60, 0.74)	< 0.001	0.78 (0.74, 0.83)	< 0.001
eGDR quartiles						
Q1	*Reference*		*Reference*		*Reference*	
Q2	0.72 (0.66, 0.78)	< 0.001	0.75 (0.64, 0.88)	< 0.001	0.71 (0.64, 0.79)	< 0.001
Q3	0.71 (0.64, 0.78)	< 0.001	0.73 (0.60, 0.89)	0.002	0.70 (0.62, 0.79)	< 0.001
Q4	0.59 (0.52, 0.66)	< 0.001	0.53 (0.42, 0.67)	< 0.001	0.60 (0.53, 0.69)	< 0.001

GDR: Q1 < 4.74, 4.74 ≤ Q2 < 6.05, Q3: 6.05 ≤ Q3 < 7.96, Q4 ≥ 7.96.

Models were adjusted for age, sex, race and ethnicity, body mass index, waist circumference, poverty income ratio, marital states, education, smoking status, alcohol consumption, physical activity.

Abbreviations: CI, confidence interval; CKM, cardiovascular-kidney-metabolic syndrome; eGDR, estimated glucose disposal rate; HR, hazard ratio.

Supplementary Table S8. Sensitivity analysis of eGDR and mortality outcomes in CKM patients after adjusting CRP

	**All-cause death**		**Cardiovascular death**		**Non-cardiovascular death**	
	**HR (95% CI)**	***P***	**HR (95% CI)**	***P***	**HR (95% CI)**	***P***
Continues eGDR	0.76 (0.73, 0.81)	< 0.001	0.70 (0.63, 0.78)	< 0.001	0.79 (0.74, 0.84)	< 0.001
eGDR quartiles						
Q1	*Reference*		*Reference*		*Reference*	
Q2	0.74 (0.67, 0.80)	< 0.001	0.79 (0.67, 0.94)	< 0.001	0.72 (0.65, 0.80)	< 0.001
Q3	0.71 (0.64, 0.79)	< 0.001	0.76 (0.62, 0.93)	0.002	0.70 (0.61, 0.79)	< 0.001
Q4	0.62 (0.55, 0.70)	< 0.001	0.61 (0.48, 0.77)	< 0.001	0.62 (0.54, 0.71)	< 0.001

GDR: Q1 < 4.74, 4.74 ≤ Q2 < 6.05, Q3: 6.05 ≤ Q3 < 7.96, Q4 ≥ 7.96.

Models were adjusted for age, sex, race and ethnicity, body mass index, waist circumference, poverty income ratio, marital states, education, smoking status, alcohol consumption, physical activity.

Abbreviations: CI, confidence interval; CKM, cardiovascular-kidney-metabolic syndrome; CRP, C-reactive protein; eGDR, estimated glucose disposal rate; HR, hazard ratio.

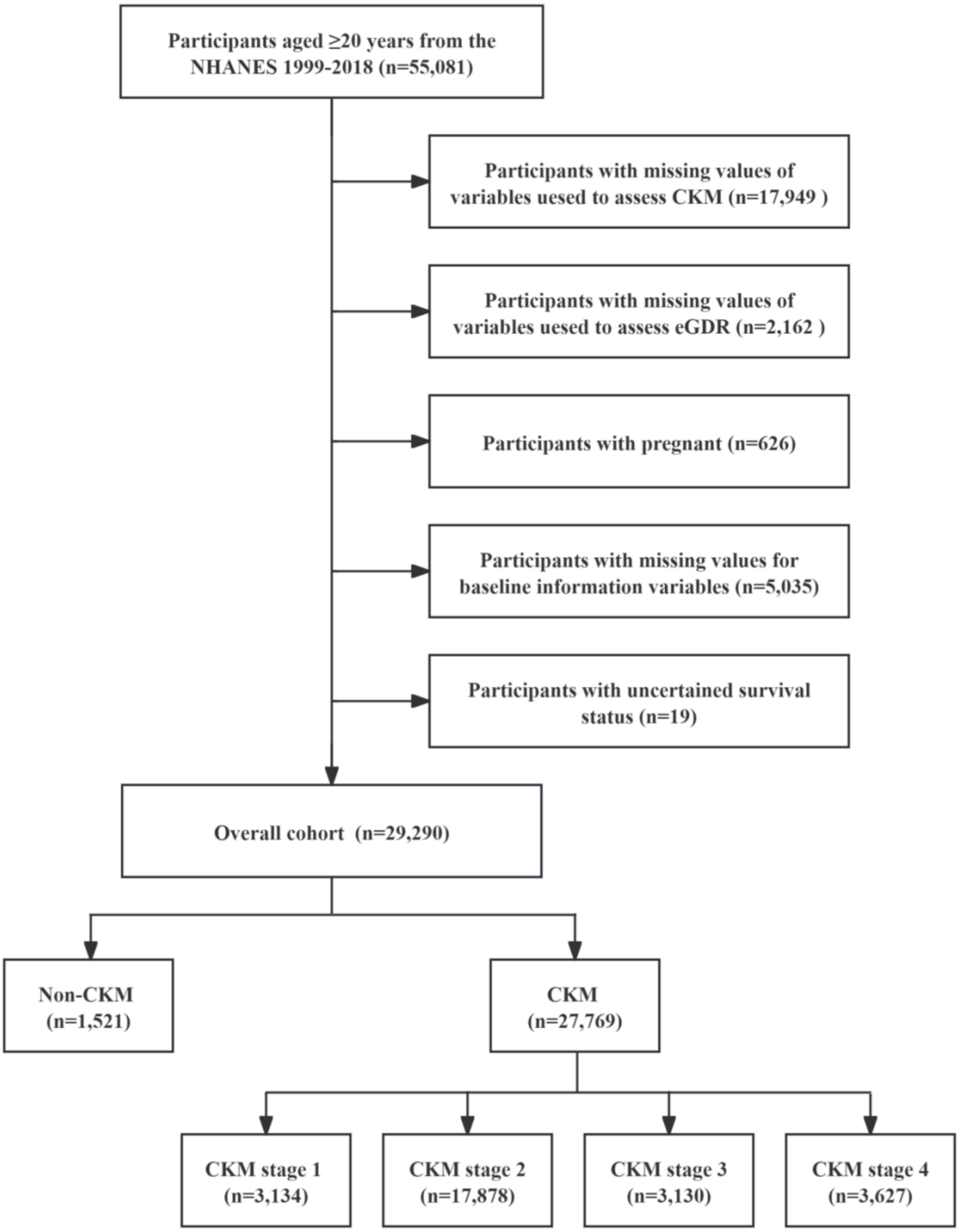

Supplementary Fig. S1 The flowchart of this study. CKM, cardiovascular-kidney-metabolic syndrome; eGDR, estimated glucose disposal rate; NHANES, National Health and Nutrition Examination Survey.

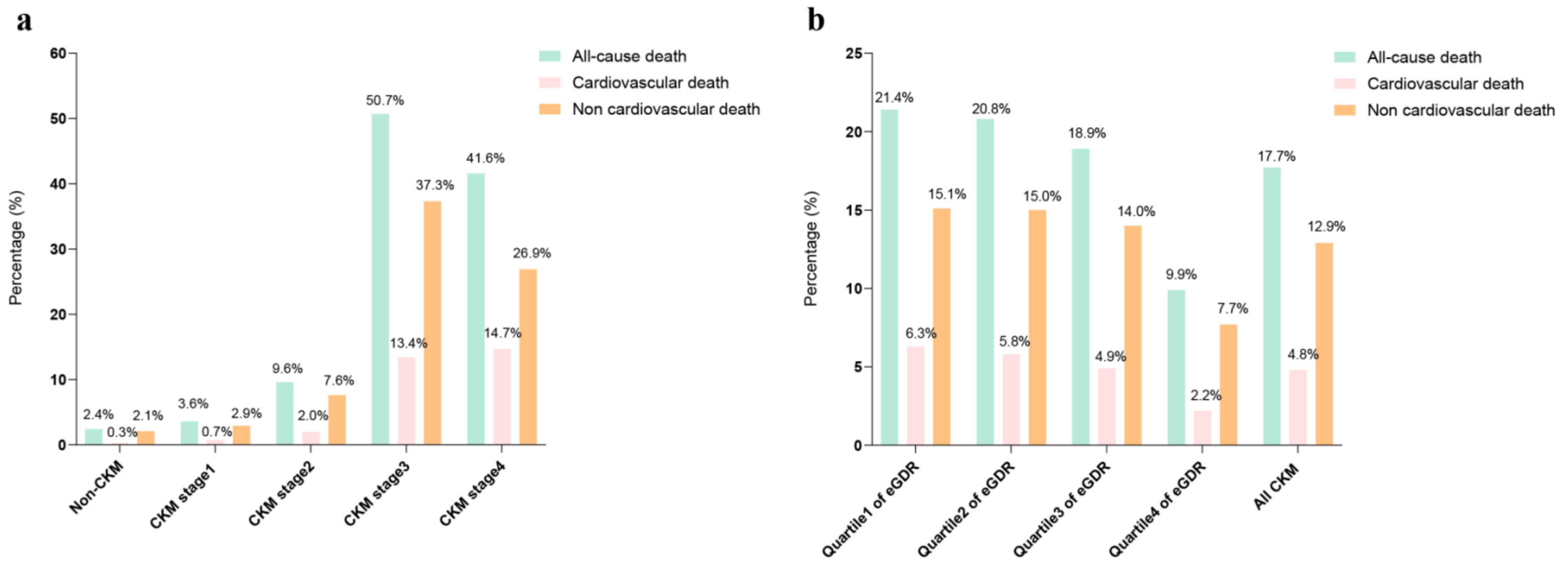

Supplementary Fig. S2 Distribution of death outcomes by CKM stages and eGDR quartiles in CKM patients. CKM, cardiovascular-kidney-metabolic syndrome; eGDR, estimated glucose disposal rate.

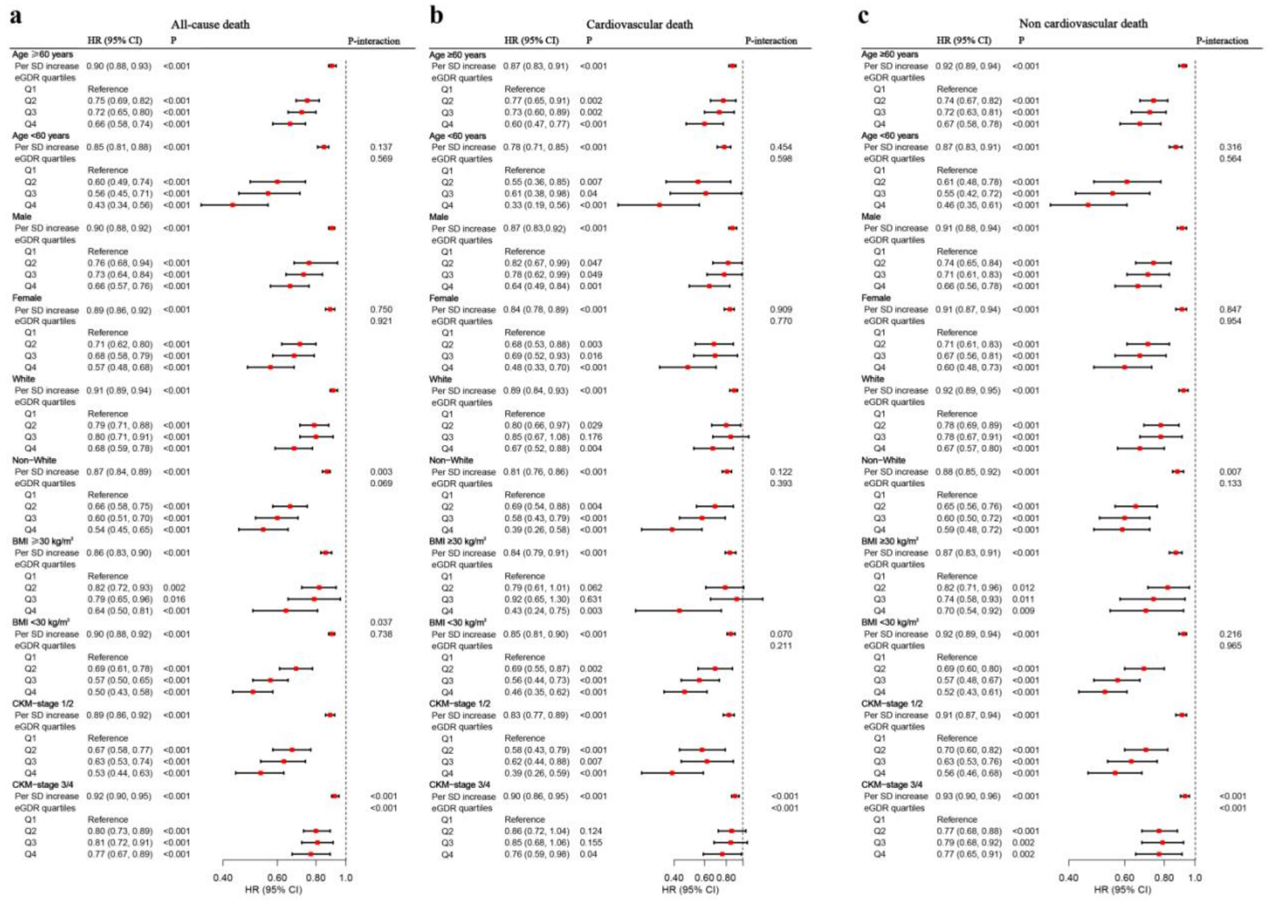

Supplementary Fig. S3 Subgroup analyses of the association between eGDR and death outcomes in CKM patients. (a) all-cause death, (b) cardiovascular death, and (c) non-cardiovascular death. BMI, body mass index; CI, confidence interval; CKM, cardiovascular-kidney-metabolic syndrome; eGDR, estimated glucose disposal rate; HR, hazard ratio.
